# The Impact of Outpatient versus Inpatient Administration of CAR-T Therapies on Clinical, Economic, and Humanistic Outcomes in Patients with Hematological Cancer: A Systematic Literature Review

**DOI:** 10.3390/cancers15245746

**Published:** 2023-12-07

**Authors:** Doris K. Hansen, Yi-Hsuan Liu, Sandip Ranjan, Hitesh Bhandari, Ravi Potluri, Lindsay McFarland, Kevin C. De Braganca, Stephen Huo

**Affiliations:** 1 H. Lee Moffitt Cancer Center & Research Institute, Tampa, FL 33612, USA; 2 Janssen Scientific Affairs, Horsham, PA 19044, USA; 3 Putnam Associates, Gurgaon 122002, India; 4 Putnam Associates, New York, NY 10282, USA; 5 Legend Biotech USA, Inc., Piscataway, NJ 08854, USA; 6 Janssen Biotech, Horsham, PA 19044, USA

**Keywords:** CAR-T, outpatient administration, effectiveness, quality of life, cost, hematological cancers, safety, outpatient monitoring, lymphoma, multiple myeloma

## Abstract

**Simple Summary:**

The administration of chimeric antigen receptor (CAR)-T cell therapies in an outpatient setting is expanding; however, there is limited evidence comparing outcomes from outpatient and inpatient administration. This research aims to compare the clinical and economic outcomes associated with the administration of CAR-T therapies in either setting for patients with hematological cancer, by summarizing existing evidence, and also to test the hypothesis that outpatient administration of CAR-T therapies yields comparable clinical effectiveness as inpatient treatment while offering significant cost reduction and lower humanistic burden.

**Abstract:**

Although chimeric antigen receptor (CAR)-T cell therapies are typically administered in the inpatient setting, outpatient administration is rapidly expanding. However, there is limited summarized evidence comparing outcomes between outpatient and inpatient administration. This systematic literature review aims to compare the safety, efficacy, quality of life (QoL), costs, and healthcare resource utilization (HCRU) outcomes in patients with hematological cancer who are administered CAR-T therapy in an outpatient versus an inpatient setting. Publications (2016 or later) that reported the outcomes of interest in patients treated with a CAR-T therapy in both outpatient and inpatient settings, or only the outpatient setting, were reviewed. In total, 38 publications based on 21 studies were included. Safety findings suggested the comparable frequency of adverse events in the two settings. Eleven studies that reported data in both settings showed comparable response rates (80–82% in outpatient and 72–80% in inpatient). Improvements in the QoL were observed in both settings while costs associated with CAR-T therapy were lower in the outpatient setting. Although unplanned hospitalizations were higher in the outpatient cohort, overall HCRU was lower. Outpatient administration of CAR-T therapy appears to have comparable outcomes in safety, efficacy, and QoL to inpatient administration while reducing the economic burden.

## 1. Introduction

Hematological cancers are a significant global health issue characterized by high mortality rates [1,2]. They negatively affect patients’ life expectancy and quality of life (QoL) and impose a substantial economic burden [3,4,5,6]. The clinical outcomes for hematological cancers have improved with the development of targeted therapies, such as small molecule inhibitors, monoclonal antibodies, and recombinant immunotoxins and, more recently, chimeric antigen receptor (CAR)-T cell therapies, antibody–drug conjugates, and bispecific T-cell engagers [7]. CAR-T therapies have emerged as a revolutionary treatment option, demonstrating remarkably effective and durable clinical responses for hematological cancers [8,9]. This therapy involves reprogramming the patient’s own T-cells to target the tumor cells wherein host T-cells are collected and are genetically modified ex vivo to express a CAR targeting a tumor-specific antigen [10]. To date, a total of six CAR-T therapies (tisagenlecleucel, axicabtagene ciloleucel, brexucabtagene autoleucel, lisocabtagene maraleucel, idecabtagene vicleucel, and ciltacabtagene autoleucel) have been approved by the United States Food and Drug Administration (FDA) for multiple hematological cancers [11,12,13] based on pivotal clinical trials demonstrating promising results of efficacy outcomes [14,15,16,17,18,19]. Notably, clinical trials focusing on CAR-T therapies have exhibited complete remission rates of 70–90% in relapsed or refractory B-cell acute lymphoblastic leukemia (ALL) [14,15,16] and 40–58% in non-Hodgkin lymphoma (NHL) [17]. Additionally, an overall response rate of 97% was shown in relapsed or refractory multiple myeloma [19,20]. Furthermore, the complete response rate of 73.1% was shown in lenalidomide-refractory multiple myeloma in a phase 3 randomized open-label trial, CARTITUDE-4 [21].

CAR-T therapies have typically been administered in an inpatient setting followed by monitoring of patients closely for several weeks for serious side effects, such as cytokine release syndrome (CRS) and neurotoxicity [22]. However, the outpatient delivery of CAR-Ts is rapidly expanding for patients with a suitable benefit–risk clinical profile and based on overall greater predictability of the clinical course and patient preference. This has the potential to significantly reduce the treatment burden for patients and caregivers and the overall cost burden to the healthcare system associated with inpatient care [22]. Previous trials have demonstrated the feasibility of outpatient CAR-T administration and indicated that such outpatient infusion may be more convenient and preferred by patients and health systems [22,23,24,25]. Challenges in outpatient CAR-T administration, however, include the availability of trained multidisciplinary teams and the infrastructure required to identify and manage complications that need early intervention, suitable reimbursement policies, and caregiver education. Furthermore, patient-specific factors including disease characteristics, clinical status, predictability of adverse events (AEs), medical center proximity, and caregiver support impact the decision on the setting of CAR-T administration [22,26]. Although there are some standalone studies that have presented a case for outpatient delivery of CAR-T therapies, there is currently no published systematic literature review (SLR) comparing the clinical safety, efficacy, QoL, economic implication, and healthcare resource utilization (HCRU) of CAR-T administration in the two settings. This SLR aims to fill this gap by identifying and summarizing the existing clinical and economic evidence on CAR-T therapies and comparing the outcomes for inpatient versus outpatient CAR-T administration in patients with hematological cancer.

## 2. Methods

### 2.1. Study Design and Search Process

This SLR was carried out in accordance with the preferred reporting items for systematic reviews and meta-analyses (PRISMA) checklist [27] and the Cochrane handbook for systematic reviews of interventions, version 6.3 [28]. The protocol has not been registered. Records were retrieved from MEDLINE, Embase, and Cochrane electronic databases. Additionally, manual searching of conference proceedings, bibliographic sources, and other grey literature sources, such as Google, Google Scholar, and disease-specific websites/conferences [American Society of Clinical Oncology (ASCO), European Society for Medical Oncology (ESMO), American Society of Hematology (ASH), European Hematology Association (EHA), International Society for Pharmacoeconomics and Outcomes Research (ISPOR), and Academy of Managed Care Pharmacy (AMCP)] was carried out. The bibliographies of relevant SLRs on the research topic were also searched to identify any additional studies. The search strategies for each database are provided in Tables S1–S3. The literature search was limited to articles published from 1 January 2016 to 4 January 2023. The starting year was chosen as 2016 to ensure the coverage of all relevant studies that may have influenced the approvals of CAR-T therapy, the first of which occurred in 2017.

### 2.2. Eligibility Criteria

This SLR included studies reporting relevant outcomes of CAR-T therapy administration in both outpatient and inpatient settings or only the outpatient setting among patients with lymphoma, ALL, or multiple myeloma. Relevant outcomes included safety, efficacy, QoL, costs, and HCRU measures. Studies that did not report the setting of CAR-T administration were excluded. Clinical trials and observational (prospective and retrospective) studies were included whereas non-human studies were excluded. The screening of articles to evaluate conformance to eligibility criteria was performed independently by two reviewers and any disagreements were resolved in discussion with a third reviewer.

### 2.3. Data Extraction

Two reviewers independently extracted the following data from the included studies: study characteristics, patient characteristics, treatment-related information, and outcomes of interest. Any inconsistencies were resolved through discussion between the two reviewers. If necessary, a third reviewer was consulted to mediate and reach a consensus.

### 2.4. Quality Assessment and Risk of Bias

The Cochrane risk-of-bias tool version 2 (RoB 2) for randomized controlled trials (RCTs) [29], the Downs and Black (Downs 1998) checklist for non-RCTs [30], and the Newcastle and Ottawa scale (NOS) for observational studies [31] were utilized for quality assessment.

### 2.5. Data Analysis

Evidence identified from the systematic literature search was analyzed qualitatively. The compiled evidence was tabulated, summarized, and presented graphically for the following elements of the research: study details (trial design, tumor type, treatment setting, sample size, and follow-up duration) and outcomes presented (safety, efficacy, QoL, HCRU, and costs incurred). Safety outcomes included the cytokine release syndrome (CRS), neurologic toxicities, and other toxicities reported in individual publications while the collated efficacy outcomes included the complete response (CR), partial response (PR), overall response rate (ORR), progression-free survival (PFS), and overall survival (OS). HCRU measures reported included the rate of, time to, and reasons for hospitalization, length of hospital stay, rate of ICU admissions and length of stay, and outpatient visits. Costs incurred in different follow-up periods post-infusion were compiled and categorized as available.

The data were reported separately for outpatient and inpatient cohorts and included evidence from comparative as well as single cohort studies.

## 3. Results

### 3.1. Literature Search Results

**Figure 1:**
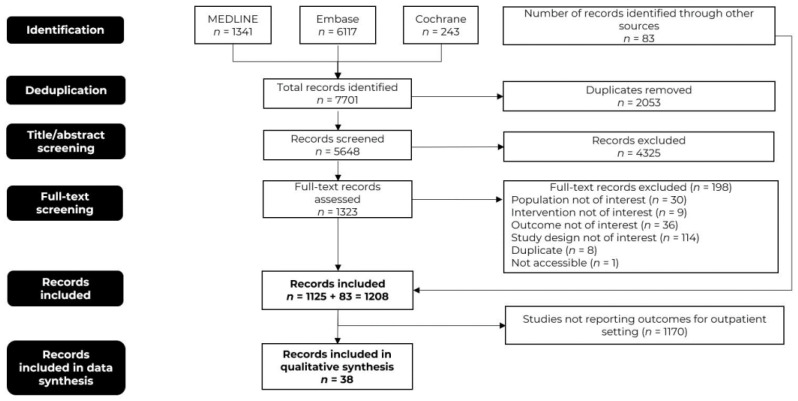
The preferred reporting items for systematic reviews and meta-analysis (PRISMA) flow diagram.

Database searches identified 7701 initial records. After deduplication, 5648 records remained for screening against inclusion and exclusion criteria. A total of 1125 records met the relevant criteria and an additional 83 records were obtained from supplementary sources including Google Scholar, conference proceedings, and a bibliography of identified studies and SLRs, resulting in a total of 1208 records. Ultimately, 38 records that reported outcomes for patients who underwent infusion/management in the outpatient setting or in both outpatient and inpatient settings were considered for qualitative synthesis (Figure 1).

### 3.2. Study Characteristics

The 38 included records were based on 21 unique studies, most of which were published in 2022 and 2023. The patient populations of these studies included individuals diagnosed with ALL, various types of lymphoma, such as B-cell lymphoma (BCL) and follicular lymphoma (FL), and multiple myeloma.

In total, 18 of these 21 studies were conducted in the United States [26,32,33,34,35,36,37,38,39,40,41,42,43,44,45,46,47,48,49,50,51,52,53,54,55,56,57,58,59,60,61,62,63]. The TRANSFORM phase 3 clinical trial was conducted in the United States, Japan, and various European countries including Belgium, France, Germany, Italy, the Netherlands, Spain, Sweden, Switzerland, and the United Kingdom [64,65]. Additionally, the ELARA phase 2 clinical trial encompassed multinational locations, including centers in the United States and Australia [66,67]. Only one retrospective study did not report the specific location where it was conducted [68]. Eleven studies reported data on CAR-T administration in both outpatient and inpatient settings [26,32,33,34,35,36,37,38,39,40,41,42,43,44,52,53,54,58,61,62,64,65,66,67,68]. These publications were based on 5 clinical trials, namely OUTREACH [32,33,34,35,36,37,38], ELARA [66,67], PILOT [39,40,41], TRANSCEND NHL 001 [42,43,44], and TRANSFORM [64,65], where the choice of site of care for patients treated with CAR-T therapies was determined at the investigator’s discretion, taking into consideration the perspectives of multiple stakeholders including healthcare providers, patients, and caregivers.

Nine of the shortlisted studies reported efficacy outcomes including response rates and survival outcomes [36,37,39,42,47,55,57,60,63,67]. Only one clinical trial reported QoL in the patient groups of interest [38]. Five studies reported data on AEs, including CRS and neurologic toxicity [37,39,42,53,67]. Seven studies reported costs/reimbursement amounts associated with the administration of CAR-T in both settings [26,41,52,53,62,65,67]. Ten studies reported data on HCRU [26,37,39,41,52,53,54,62,67,68] in both settings while a further seven studies reported HCRU among patients who received CAR-T only in the outpatient setting [26,37,39,41,46,52,53,54,55,57,59,60,62,63,64,67,68]. The distribution of age, sex, performance status, number of prior treatment lines, and other patient characteristics varied across the studies. Details of the included studies and patient characteristics are shown in Table 1 and Table 2.

### 3.3. Quality Assessment

All of the 21 studies included in the analysis underwent a quality assessment using relevant checklists based on their study design. The assessment aimed to evaluate the risk of bias and the overall quality of the studies. The ROB 2 checklist was used to assess the RCTs conducted by Kamdar in 2022 [64]. The findings from this assessment revealed a high risk of bias in the trial (Table S4). The modified Downs and Black checklist with 27 items assessed eight non-randomized single-arm trials. Table S5 highlights one study of good quality (score, 15–17), five of fair quality (score, 12–14), and two of poor quality (score < 11) [38,39,42,46,49,51,67]. The NOS criteria were used to assess the quality of double-arm and single-arm observational studies. Of the six double-arm studies, five were considered of good quality (score > 6) and one of fair quality (score 5) (Table S6). Among the six single-arm studies, five were rated as good quality (score > 4) and one as fair quality (score 4) [26,41,45,53,54,55,57,59,60,62,63,68].

### 3.4. Clinical Outcomes

#### 3.4.1. Safety

The safety of CAR-T therapy was evaluated in several studies, with data available for both outpatient and inpatient settings [37,39,42,53,67]. In total, 5 publications provided safety data for both settings while 10 publications reported data solely on patients treated in the outpatient setting. In the publications reporting data for both settings, the frequency of AEs was generally comparable or higher for patients who received CAR-T in the inpatient setting. Two publications by Denlinger (2022) [53] and Sehgal (2022) [39] reported a higher frequency of CRS in patients who received CAR-T in the inpatient setting whereas the other three publications found similar rates of CRS between the two cohorts. The OUTREACH and ELARA trials reported other AEs, including infections, leukopenia, anemia, and thrombocytopenia. In the OUTREACH trial, the incidence of infections was comparable between the outpatient and inpatient settings, with 33% and 32% of patients experiencing infections, respectively. Additionally, prolonged cytopenia at the Day 29 visit was also consistent between outpatient and inpatient settings, with 33% and 32% of patients affected, respectively. In the ELARA trial, the incidence of infections was lower in the outpatient setting compared with the inpatient setting, with any grade infections reported in 17.6% of outpatient versus 21.3% of inpatient cohorts. Although none of the patients in the outpatient group experienced a Grade 3–4 infection, 7.5% of those in the inpatient group experienced such severe infections. Furthermore, the ELARA trial indicated a higher occurrence of hematological disorders in the inpatient setting, with any grade cytopenia reported in 77.5% of inpatient compared with 64.7% of outpatient administration groups. Similarly, the occurrence of Grade 3–4 cytopenia was lower in the outpatient group (47.1%) versus the inpatient group (73.8%). Three publications reported a higher frequency of neurologic toxicities among patients who received CAR-T in the inpatient setting [39,53,67]. Conversely, one publication by Abramson (2020) [42] reported a substantially higher frequency of neurologic toxicities among patients who received CAR-T in the outpatient setting (Table 3). Most of the publications pertaining to only the outpatient cohort reported high frequencies of CRS-related (40–92%) and neurologic (6–57%) toxicities [45,47,48,49,51,55,57,59,60,63]. Detailed safety outcomes are presented in Table 4. For studies involving CAR-T treatment in both inpatient and outpatient settings, three publications reported no deaths from CRS and neurological toxicities [37,39,42]. For studies based on treatment in only the outpatient setting, Turtle et al. reported a 5% mortality rate, with most deaths occurring during the dose-finding phase, primarily due to CRS and neurological events [51].

#### 3.4.2. Efficacy: Response and Survival Outcomes

In the studies reporting response outcomes from both settings, the overall response rate (ORR) was found to be comparable between patients managed in the outpatient setting (ORR, 80–82%) and those managed in the inpatient setting (ORR, 72–80%) [36,37,39,42]. In the OUTREACH trial, the ORR was 82% in the outpatient cohort and 76% in the inpatient cohort [36,37]. In the PILOT trial, the ORR was 80% in both outpatient and inpatient groups [39]. In the TRANSCEND NHL 001 trial, the ORR was 80% in the outpatient cohort and 72% in the inpatient cohort [42]. The response rates observed in real-world studies (43–88%) were lower than those observed in clinical trials (80–100%) for patients managed in the outpatient setting. The median duration of response was found to range from 8.2 to 15.1 months among the CAR-T–treated patients managed in the outpatient setting and were comparable to the duration of response in those managed in the inpatient setting (12.1 to 14.8 months) (Table 5) [36,37,39]. Progression-free survival (PFS) data for patients managed in the outpatient setting were reported in a limited number of studies. In the OUTREACH trial, patients receiving treatment in the outpatient setting had a 12-month PFS rate of 41% which was similar to the rate observed in an inpatient setting (39%). In the ELARA trial, the 12-month PFS rate was 60% for patients in the outpatient group compared with 70% for patients receiving treatment in an inpatient setting (Table 6) [37,39,42,63,67]. Similarly, the overall survival (OS) data for patients managed in the outpatient setting were limited. The Kaplan–Meier estimates for 12-month OS ranged from 60% to 75% among patients with lymphoma managed in the outpatient setting and were reported to be 60% among those managed in the inpatient setting [37,39,47,60,63]. Notably, a real-world study reported a median OS of 26.5 months in patients with lymphoma managed in the outpatient setting (Table 7) [60].

#### 3.4.3. Quality of Life

Linhares et al. (2022) reported QoL assessments in patients treated with CAR-T therapy at multiple time points, including pre-treatment (baseline), on day of infusion, on several dates after infusion (days 29, 50, 90, 180, 270, 365, 545, and 730/end of study), and at disease progression and assessed the least squares (LS) mean change from the baseline for visits with ≥10 patients. Both the outpatient and inpatient groups exhibited comparable meaningful improvements in various aspects of QoL, including global health status/QoL, fatigue, pain, assessed by EORTC-QLQ-C30, and the EuroQol 5-Level 5-Dimension questionnaire (EQ-5D-5L) visual analog scale (VAS) scores (Table 8) [38].

### 3.5. Economic Outcomes

#### 3.5.1. Direct Costs

Seven studies reported costs/reimbursement amounts associated with CAR-T administration in both outpatient and inpatient settings in the United States (Table 9) [26,41,52,53,62,65,67]. Of these, six reported that the post-infusion costs were lower for patients who received CAR-T in an outpatient setting compared with those who received it in an inpatient setting [41,52,53,62,65,67]. Costs in the 6-month post-infusion period reported from TRANSCEND NHL 001, OUTREACH, TRANSFORM, and PILOT trials revealed to be two to four times greater costs in the inpatient setting—these ranged from USD 61,772 to USD 96,297. Pooled analysis of TRANSCEND NHL 001 and OUTREACH trials similarly showed a lower 6-month post-infusion cost in the outpatient setting (USD 36,702) compared with the inpatient setting (USD 89,535) [52]. The main driver for the higher costs in the inpatient setting was identified to be hospitalization costs [41,52,65]. Yang et al. (2022), the only study that assessed the Medicare reimbursement amount, including CAR-T product costs, reported that costs in the first month were nominally higher in the outpatient cohort compared with the inpatient cohort and were comparable in subsequent months [26].

#### 3.5.2. Healthcare Resource Utilization

The analysis of HCRU among patients who received CAR-T in the outpatient setting was reported in 17 publications [26,37,39,41,46,52,53,54,55,57,59,60,62,63,64,67,68]. Of these, 10 also reported data for patients who received CAR-T in the inpatient setting (Table 10) [26,37,39,41,52,53,54,62,67,68]. Most of the HCRU data were related to inpatient admissions, length of stay (LOS), and intensive care unit (ICU) admissions. Some publications also reported a median time to hospitalization for the outpatient cohort, ranging from 4 to 9 days [37,39,52,55,57,64,67]. Chihara et al. (2022) and Wright et al. (2020) reported lower rates of unplanned hospitalization (defined as post-CAR-T infusion in the outpatient cohort and re-hospitalizations in the inpatient cohort) in the inpatient cohort [54,68]. Despite such higher unplanned hospitalization observed in the outpatient cohort, the overall HCRU was lower for the outpatient cohort in publications that reported data for both cohorts. Most studies reported higher overall mean LOS for the inpatient cohort, with two to three times longer durations compared with the outpatient cohort across studies [26,37,39,41,52,53,62,67]. Additionally, ICU utilization was found to be higher for the inpatient vs. outpatient cohort, as shown by Fowler et al. (2022) (9% vs. 0%), Palomba et al. (2020) (7% vs. 6%), and Sehgal et al. (2022) (20% vs. 5%) [26,41,52,53,62,65,67]. Similarly, the mean ICU LOS was two to three times higher for the inpatient cohort across studies, except the study by Sehgal et al. (2022) which reported a lower ratio (0.8) of ICU LOS for the inpatient cohort [39]. Additional HCRU data available in the identified studies are presented in Table S7.

## 4. Discussion

This comprehensive SLR on CAR-T therapies in patients with hematological cancer highlights the potential benefits of outpatient compared with inpatient administration. Safety outcomes, a key consideration in CAR-T treatments and a principal driver for traditionally treating patients in the inpatient setting, were comparable between those treated in the outpatient setting and those treated in the inpatient setting. Moreover, the analysis revealed comparable effectiveness outcomes between the two settings, including the response rates, duration of response, and survival outcomes where reported. Furthermore, both outpatient and inpatient cohorts experienced meaningful improvements in QoL measures. These findings collectively provide compelling evidence to clinicians and other decision-makers to actively consider administering CAR-T therapies in the outpatient setting for patients whose disease and clinical characteristics permit this. Treating institutions may upgrade their processes and protocols to encourage treatment in the outpatient setting, including preparing for and managing early complications.

In the reviewed studies, the choice of outpatient administration was at the investigator’s discretion, taking into account patient disease characteristics, clinical status, and logistical considerations, such as the availability of caregiver support and the ability to remain within a short distance from the treatment site for 30 days after infusion [32,34,35,36,37,38,39,42,43,44,64,65,66,67]. Typically, patients receiving CAR-T therapy in the outpatient setting are closely monitored by a multidisciplinary CAR-T therapy team and adhere to standard operating procedures for outpatient AE monitoring and management [32,34,35,36,37,38,66,67].

To further bolster the case for outpatient administration of CAR-T therapies, assessment of such administration in the real world with longer-term follow-up to allow for the evaluation of survival outcomes such as PFS and OS is warranted. These endpoints are crucial in evaluating the long-term benefits and potential risks associated with the two treatment settings [69].

The observed enhancement in QoL reported by Linhares et al. (2022) likely represents the comprehensive impact of CAR-T therapy on hematological cancer patients, regardless of the treatment setting [38]. Further research is needed to explore the underlying factors contributing to the improvement in QoL, paying particular attention to factors related to reduced hospitalization as this could enhance the overall patient experience and improve their QoL during treatment. It is also important to evaluate QoL at multiple time points after CAR-T therapy use and examine if the resolution of AEs reflects improved QoL outcomes. Furthermore, the utilization of other measures, such as the hospital anxiety and depression scale depression subscale (HADS-D), in patients with hematological cancer is important in understanding and addressing their mental health needs more specifically [70]. Additionally, a previous SLR emphasized the importance of identifying patients’ preferences for involvement in cancer treatment decisions. Establishing these preferences will encourage the healthcare system to become more responsive to individual patient needs and expectations and ultimately, contribute to improving their QoL [71].

The economic outcomes indicate that outpatient treatment may offer cost advantages over inpatient treatment, with patients treated in the latter incurring two to four times higher costs (USD 62,000–96,000) than by outpatient-treated patients (USD 16,000–38,000) [41,52,65]. The lower costs observed in the outpatient cohort were primarily driven by reduced hospitalization costs [41,62,65,67]. These findings align with an economic evaluation of CAR-T therapy based on the site of care, wherein outpatient CAR-T administration resulted in a substantial decrease in total costs (by 40.4%), with notable reductions observed in hospitalization, office visits, and procedural expenses [72]. However, a study by Yang (2022) found that the Medicare reimbursement in the outpatient cohort was slightly higher than that in the inpatient cohort during the first month post-infusion and comparable in subsequent months. The authors, however, attributed this to the inadequate reimbursement for CAR-T infusion in the inpatient setting whereas the outpatient setting reimbursement, which is covered under Medicare Part B, covers not only the CAR-T product cost more completely but also the handling, storage, and a portion of the physician’s service fees. Additional efforts are recommended to improve the reimbursement structure and care policies for CAR-T therapy in either infusion setting to suitably incentivize providers [26].

The outpatient cohort experienced more unplanned hospitalizations, with CRS being the main reason for hospitalization. However, the overall HCRU was lower in cases where data were available for both settings as the inpatient cohort exhibited longer stays and a higher HCRU [26,37,39,41,46,52,53,54,55,56,57,59,60,62,63,64,67,68]. An improved understanding of the predictive risk factors for CRS and neurotoxicity development, including patient disease characteristics and clinical status, can influence personalized decisions regarding outpatient administration as it may offer potential benefits in terms of overall resource utilization.

Outpatient CAR-T administration can potentially expand treatment access by freeing up inpatient capacity and addressing geographic obstacles. A prior economic model concluded that lower costs through outpatient administration could enable more patients to receive treatment with limited resources. [72].

This review provides a comprehensive analysis of both clinical and economic outcomes derived from clinical trials and observational studies concerning CAR-T therapies in a broad patient population with hematological cancer. The review was conducted in accordance with a predefined protocol, with clear inclusion and exclusion criteria, and adhered to the Cochrane guidelines for systematic review reporting. A comprehensive search strategy was employed to minimize reporting bias in the review process.

While this SLR adhered to rigorous selection criteria, it had some limitations. The included studies exhibited heterogeneity in terms of methodology and populations, which prevented direct comparisons. Additionally, in studies that reported outcomes related to the two settings, patients were not randomized between the settings, introducing the potential for bias and raising concerns about the comparability of reported outcomes. In line with the objective of this study, outcomes data were presented here only if reported by setting. The molecular aspect of the therapy has not been discussed as none of the identified publications referred to it as either the driver for the decision of inpatient vs. outpatient administration or as the cause of any difference in the outcomes. Furthermore, patient characteristics were not available for all the studies as the majority of the identified publications were conference abstracts with minimal information on patient characteristics.

## 5. Conclusions

Findings from our study showed comparable overall outcomes in safety, efficacy, and QoL between outpatient and inpatient CAR-T administration. While CAR-Ts are typically administered in an inpatient setting, outpatient administration of CAR-T can provide a reduced economic burden without negatively impacting clinical outcomes and should be actively considered where patient disease characteristics and logistical considerations permit this. Future research is needed to explore the impact of administration settings of new CAR-T therapies on patients with multiple myeloma and other hematological cancers.

## Figures and Tables

**Table 1:** Characteristics of included studies.

Study	Country	Trial Name/ID	Study Design	Treatment	Patient Population	Setting (IP/OP/Both)	Sample Size, *n*	Reason for Different Sample Sizes	List of Outcomes
Kamdar (2022) [64]	US, Europe, and Japan	TRANSFORM/NCT03575351	Ph 3 trial	Liso-cel	R/R LBCL (2L+)	Both	91		Response, LOS
McGarvey (2022) [65]							90		Total post-infusion monitoring costs, hospitalizations cost
Godwin (2021) [32]	US	OUTREACH/NCT03744676	Ph 2 trial	Liso-cel	R/R LBCL (3L+)	Both	34	Preliminary results 1	Response, AEs, hospitalizations, LOS
Godwin (2020) [33]							34		Response, AEs, hospitalizations, time to hospitalization, LOS
Godwin (2021) [34]							46	Preliminary results 2	Response, AEs
Godwin (2021) [35]							46		Response, AEs, hospitalizations, time to hospitalization, LOS
Godwin (2021) [36]							71	Preliminary results 3	Response, AEs, time to hospitalization, LOS
Linhares (2022) [37]							82	Updated results	Response, AEs, hospitalizations, time to hospitalization
Linhares (2022) [38]							82		Response, AEs, hospitalizations, time to hospitalization, QoL
Sehgal (2022) [39]	US	PILOT/NCT03483103	Ph 2 trial	Liso-cel	R/R LBCL (2L+ not intended for HSCT)	Both	61		Response, time to response, DOR, PFS, OS, EFS, AEs, hospitalization, ICU admission
Sehgal (2019) [40]					R/R aggressive NHL (2L+)		10	Subgroup	Response, AEs, HCRU
McGarvey (2022) [41]					R/R LBCL (2L+ not intended for HSCT)		61		Hospitalizations, LOS, monitoring costs
Abramson (2020) [42]	US	TRANSCEND NHL 001/NCT02631044	Ph 1 trial	Liso-cel	R/R LBCL (2L+)	Both	25	Preliminary results 1	Response, PFS, AEs
Maloney (2017) [43]					R/R B-cell NHL (2L+)		69	Preliminary results 2	AEs
Palomba (2018) [44]					R/R DLBCL (2L+)		94	Updated results	Response, AEs, hospitalizations, time to hospitalization, ICU admission, LOS, OP visits
Gofshteyn (2018) [45]	US	Pedi CART19/NCT01626495	Ph 1/2a trial	Tisa-cel	Pediatric and young adults with R/R ALL	OP	51	NA	AEs
Fowler (2021) [66], Fowler (2023) [67]	Multinational	ELARA/NCT03568461	Ph 2 trial	Tisa-cel	R/R FL (3L+)	Both	97	NA	Hospitalizations, LOS, ICU admission, hospitalization costs
Myers (2020) [46]	US	NCT01626495/NCT02906371/NCT02374333	Pooled analysis (3 Ph 1/2 trials)	Tisa-cel	Pediatric ALL	93% patients treated as OPs	213	NA	Hospitalizations, LOS, ICU admission, mortality rate, other HCRU
Shadman (2021) [47]	US	NCT03277729	Ph 1/2 trial	MB-106	R/R B-NHL (FL, MCL, DLBCL) and CLL	OP	25	Updated results	Response, AEs
Shadman (2020) [48]							12	Preliminary results	
Shadman (2021) [49]							12		
Shadman (2022) [50]					R/R FL		16	Updated results for a subgroup	
Turtle (2017) [51]	US	NCT01865617	Ph 1/2 trial	CD19 CAR-T cells	B-ALL, NHL or CLL	OP	161	NA	AEs
Palomba (2020) [52]	US	-	Cost analysis (pooled trial data)	Liso-cel	R/R LBCL (3L+)	Both	303	NA	Standard IP and ICU LOS, costs (diagnostics and procedures, medications, hospitalization/ICU)
Denlinger (2022) [53]	US	-	Retrospective study	Tisa-cel, axi-cel	BCL	Both	63	NA	AEs, LOS, costs and charges, out-of-pocket charges
Chihara (2022) [54]	US	-	RWE	Any CAR-T	R/R DLBCL	Both	551	NA	PFS, OS, hospitalization, ER visits, OP visits (initial and follow-up), costs
Borogovac (2022) [55], Borogovac (2021) [56]	US	-	Retrospective study	Any CAR-T	DLBCL, FL and ALL, MCL	91% patients treated as OPs	23	NA	Response, AEs
Shao (2021) [57]	US	-	Retrospective study	Tisa-cel	DLBCL (3L+)	OP	12	NA	Response, AEs, hospitalization, LOS
Yang (2022) [26], Zhao (2021) [58]	US	-	RWE (Medicare claims database)	Tisa-cel, axi-cel	R/R DLBCL	Both	430	NA	ICU admission, LOS, costs
Farooqui (2022) [59]	US	-	Retrospective study	Axi-cel	NHL	OP	83	NA	AEs, ICU admission
Nasta (2022) [60]	US	-	Retrospective study	Tisa-cel	Lymphoma (3L+)	OP	72	NA	Response, OS, PFS, AEs
Maziarz (2021) [61] Maziarz (2022) [62]	US	-	Retrospective study	Tisa-cel, axi-cel	R/R DLBCL	Both	119	NA	Hospitalizations, ICU admissions, LOS, OP visits, costs
									Hospitalizations, ICU, LOS, OP visits (initial and follow-up), costs
Wright (2020) [68]	NR	-	Retrospective study	Axi-cel/tisa-cel	R/R NHL	Both	31	NA	Hospitalizations, LOS
Kirby (2022) [63]	US	-	Retrospective study	Axi-cel, tisa-cel, brexu-cel, liso-cel	R/R BCL	OP	20	NA	PFS, OS, AEs, hospitalizations

Abbreviations: 2L+, second line and greater; 3L+, third line and greater; AE, adverse event; ALL, acute lymphoblastic leukemia; axi-cel, axicabtagene ciloleucel; brexu-cel, brexucabtagene autoleucel; BCL, B-cell lymphoma; CAR-T, chimeric antigen receptor T-cell; CLL, chronic lymphocytic leukemia; DLBCL, diffuse large B-cell lymphoma; DOR, duration of response; EFS, event-free survival; ER, emergency room; FL, follicular lymphoma; HCRU, healthcare resource utilization; HSCT, hematopoietic stem cell transplant; ICU, intensive care unit; IP, inpatient; LBCL, large B-cell lymphoma; liso-cel, lisocabtagene maraleucel; LOS, length of stay; MCL, mantle cell lymphoma; NA, not available; NHL, non-Hodgkin lymphoma; NR, not reported; OP, outpatient; OS, overall survival; Ph, phase; PFS, progression-free survival; PR, partial response; QoL, quality of life; R/R, relapsed/refractory; RWE, real-world evidence; tisa-cel, tisagenlecleucel; US, United States.

**Table 2:** Patient characteristics.

Study	Study/Trial Name/Trial ID	Treatment	Setting (IP/OP)	*n*	Age, Median (Years)	Age ≥ 65, (%)	Male (%)	ECOG PS (%)	Number of Prior Lines	Prior Transplant Therapy, (%)	Refractory Patients, (%)
Godwin (2021) [36]	OUTREACH/NCT03744676	Liso-cel	OP	54	64.5	50.0	70.0	0: 33.0 1: 67.0	2.0 (2.0–4.0) *	20.0	89.0
			IP	23	68.0	61.0	61.0	0: 26.0 1: 74.0	2.0 (1.0–6.0) *	9.0	96.0
Fowler (2021) [66], Fowler (2023) [67]	ELARA/NCT03568461	Tisa-cel	OP	17	NR	23.5	76.5	≥1: 23.5	≥5: 41.2%	NR	41.2
			IP	80	NR	25.0	63.8	≥1: 47.5	≥5: 25.0%	NR	26.3
Gofshteyn (2018) [45]	Pedi CART19—NCT01626495	Liso-cel	OP	51	11.5	NR	49.0	NR	NR	NR	NR
Shadman (2022) [50]	NCT03277729	MB-106 (CD20 CART-T)	OP	16	61.5	NR	NR	NR	NR	NR	NR
Myers (2020) [46]	NCT01626495/NCT02906371/NCT02374333	Tisa-cel	OP	213	12.4	NR	60.0	NR	NR	NR	NR
Shao (2021) [57]	NR	Tisa-cel	OP	12	69.5	83.0	66.7	NR	2: 75.0% 3: 25.0%	16.7	NR
Zhao (2021) [58], Yang (2022) [26]	NR	Tisa-cel, axi-cel	OP	50	Mean: 68.4	NR	70.0	NR	NR	NR	NR
			IP	380	Mean: 70.8	NR	62.4	NR	NR	NR	NR
Farooqui (2022) [59]	NR	Axi-cel	OP	83	Mean: 55.2	NR	65.1	NR	NR	NR	NR
Nasta (2022) [60]	NR	Tisa-cel	OP	72	65.7	NR	58.3	0: 31.9 1: 62.5 2: 4.2 NR: 1.4	NR	18.1	NR
Kirby (2022) [63]	NR	Tisa-cel, axi-cel, liso-cel, brexu-cel	OP	20	69.5	NR	60.0	≥2: 25.0	NR	NR	NR

* Median (range); Abbreviations: axi-cel, axicabtagene ciloleucel; brexu-cel, brexucabtagene autoleucel; CAR-T, chimeric antigen receptor T-cell; ECOG PS, Eastern Cooperative Oncology Group performance status; IP, inpatient; liso-cel, lisocabtagene maraleucel; NR, not reported; OP, outpatient; tisa-cel, tisagenlecleucel.

**Table 3:** Safety data in inpatient and outpatient settings reported in the included studies.

First Author, Year	Trial Name/ID	Study Design	Patient Population	Treatment	Site of Care	Follow-Up	*n*	CRS-Related Toxicity (%)	Neurologic Toxicity (%)	Other AEs (%)
Linhares (2022) [37]	OUTREACH/NCT03744676	Ph 2 trial	R/R LBCL (3L+)	Liso-cel	Outpatient vs. inpatient	NA	54 vs. 25	Any grade: 37.0 vs. 44.0	Any grade: 30.0 vs. 32.0 Grade 3–4: 13.0 vs. 4.0	Infection: Any grade, 33 vs. 32; Grade 3–4, 13.0 vs. 4.0. Prolonged cytopenia at Day 29 visit: Any grade, 33.0 vs. 32.0. Hypogammaglobulinemia: Any grade, 11.0 vs. 4.0
Fowler (2023) [67]	ELARA/NCT03568461	Ph 2 trial	R/R FL (3L+)	Tisa-cel	Outpatient vs. inpatient	Median: 20 months	17 vs. 80	Any grade: 52.9 vs. 47.5	Any grade: 5.9 vs. 11.3 Grade 3–4: 0.0 vs. 1.3	At least 1 AE: Any grade, 82.4 vs. 92.5; Grade 3–4, 47.1 vs. 75. Hematological disorders, including cytopenias: Any grade, 64.7 vs. 77.5; Grade 3–4, 47.1 vs. 73.8. Infections: Any grade, 17.6 vs. 21.3; Grade 3–4, 0.0 vs. 7.5. Prolonged depletion of normal B cells or agammaglobulinemia: Any grade, 11.8 vs. 10.0. Tumor lysis syndrome: Any grade: 0.0 vs. 1.3; Grade 3–4, 0.0 vs. 1.3
Sehgal (2022) [39]	PILOT/NCT03483103	Ph 2 trial	R/R LBCL (2L+ not intended for HSCT)	Liso-cel	Outpatient vs. inpatient	Median: 12.3 months	20 vs. 41	Any grade: 15.0 vs. 48.0	Any grade: 10.0 vs. 41.0	NA
Abramson (2020) [42]	TRANSCEND NHL 001/NCT02631044	Ph 3 trial	R/R LBCL (2L+)	Liso-cel	Outpatient vs. inpatient	Median: 18.8 months	25 vs. 244	Any grade: 40.0 vs. 42.0 Grade 3–4: 4.0 vs. 2.0	Any grade: 44.0 vs. 28.0 Grade 3–4: 8.0 vs. 10.0	NA
Denlinger (2022) [53] *	NA	Retrospective study	BCL	Tisa-cel vs. axi-cel	Outpatient vs. inpatient	NA	18 vs. 45	Any grade: 44.0 vs. 96.0	Any grade: 22.0 vs. 73.0	NA

* Denlinger et al. did not specify the settings; however, it seems that tisa-cel was administered in the outpatient setting and axi-cel in the inpatient setting; Abbreviations: 2L+, second line and greater; 3L+, third line and greater; AE, adverse event; axi-cel, axicabtagene ciloleucel; BCL, B-cell lymphoma; CRS, cytokine release syndrome; FL, follicular lymphoma; HSCT, hematopoietic stem cell transplant; LBCL, large B-cell lymphoma; liso-cel, lisocabtagene maraleucel; NA, not available; Ph., phase; R/R, relapsed/refractory; tisa-cel, tisagenlecleucel.

**Table 4:** Safety data in outpatient settings reported in the included studies.

First Author, Year	Trial Name/ID	Study Design	Patient Population	Treatment	Follow-Up	Sample Size, *n*	CRS-Related Toxicity (%)	Neurologic Toxicity (%)	Other AE (%)
Gofshteyn (2018) [45]	Pedi CART19/NCT01626495	Ph 1/2a trial	Pediatric and young adult patients with R/R ALL	Tisa-cel	NA	51	Any grade: 92.0	Any neurotoxicity: 45.0 Common neurotoxicity: 41.0	NA
Turtle (2017) [51]	NCT01865617	Ph 1/2 trial	B-ALL, NHL or CLL	Anti-CD19 CAR-T cell therapy	NA	133	Any grade: 71.0 Grade 1–2: 60.0 Grade 3: 4.0 Grade ≥ 4: 8.0	Any grade: 40.0 Grade 1–2: 19.0 Grade 3: 16.0 Grade ≥ 4: 5.0	NA
Shadman (2021) [49]	NCT03277729	Ph 1/2 trial	R/R B-NHL and CLL (FL, MCL, CLL, DLBCL) Entire cohort	CD20 CAR-T cells	NA	12	Grade 1: 16.0 Grade 2: 8.0	NA	NA
Shadman (2021) [47]	NCT03277729	Ph 1/2 trial	FL	CD20 Targeted CAR T-cell therapy (MB-106)	Maximum 13.0 months post-infusion	18	Grade 1: 22.0 Grade 2: 5.5 Grade 3: 0.0 Grade 4: 0.0	Grade 1: 0.0 Grade 2: 0.0 Grade 3: 0.0 Grade 4: 0.0	NA
			CLL, MCL, DLBCL, WM			7	Grade 1: 28.0 Grade 2: 28.0 Grade 3: 0.0 Grade 4: 0.0	Grade 1: 14.0 Grade 2: 14.0 Grade 3: 0.0 Grade 4: 0.0	NA
			FL, CLL, MCL, DLBCL, WM			25	Grade 1: 24.0 Grade 2: 12.0 Grade 3: 0.0 Grade 4: 0.0	Grade 1: 4.0 Grade 2: 4.0 Grade 3: 0.0 Grade 4: 0.0	NA
Shadman (2020) [48]	NCT03277729	Ph 1/2 trial	R/R B-cell NHL	CD20 CAR-T	NA	11	NA	NA	Grade ≥ 3 AEs Anemia: 36.0 Lymphopenia: 27.0 Neutropenia: 18.0 Hypertension: 9.0 Hypotension: 9.0 Thromboembolic event: 9.0 Neutropenia: 9.0 Elevated alkaline phosphatase: 9.0 Pneumonia: 9.0 Bacteremia: 9.0 Hyperglycemia: 9.0 Pleural effusion: 9.0 Generalized pain: 9.0
Shao (2021) [57]	NA	Retrospective study	DLBCL	Tisa-cel	Median: 29.1 weeks, (range: 2.6–60.0)	12	Any grade: 50.0 Grade 3–4: 8.3	Any grade: 8.3 Grade 3–4: 0.0	Any grade AEs Anemia: 75.0 Thrombocytopenia: 66.7 Neutropenia: 66.7 Grade 3–4 AEs Anemia: 33.3 Thrombocytopenia: 37.5 Neutropenia: 66.7
Borogovac (2022) [55]	NA	Retrospective study	DLBCL, ALL, MCL	Axi-cel	NA	13	Any grade: 69.0 Grade 3–4: 8.0	Any grade: 31.0 Grade 3–4: 0.0	NA
				Tisa-cel	NA	6	Any grade: 50.0 Grade 3–4: 17.0	Any grade: 17.0 Grade 3–4: 17.0	NA
				Brexu-cel	NA	1	Any grade: 0.0 Grade 3–4: 0.0	Any grade: 100 Grade 3–4: 0.0	NA
				Liso-cel	NA	1	Any grade: 0.0 Grade 3–4: 0.0	Any grade: 0.0 Grade 3–4: 0.0	NA
Farooqui (2022) [59]	NA	Retrospective study	Refractory NHL, patients without acute kidney injury	Axi-cel	NA	69	Incidence: 85.5 Grade None: 14.5 Grade 1: 49.3 Grade 2: 34.8 Grade 3: 0.0 Grade 4: 1.4	Incidence: 52.2 Grade None: 47.8 Grade 1: 21.7 Grade 2: 18.8 Grade 3: 7.2 Grade 4: 4.3	NA
			Refractory NHL, patients with acute kidney injury	Axi-cel	NA	14	Incidence: 85.7 Grade None: 14.3 Grade 1: 35.7 Grade 2: 42.9 Grade 3: 0.0 Grade 4: 7.1	Incidence: 57.1 Grade None: 42.9 Grade 1: 7.1 Grade 2: 21.4 Grade 3: 21.4 Grade 4: 7.1	NA
Nasta (2022) [60]	NA	Retrospective study	Lymphoma (3L+)	Tisa-cel	Median: 39.5 weeks (range: 3.0–127.3)	72	None: 59.7 Grade 1: 22.2 Grade 2: 18.1	None: 94.4 Grade 1: 2.8 Grade 3–4: 2.8	NA
Kirby (2022) [63]	NA	Retrospective study	R/R BCL	Tisa-cel, axi-cel, liso-cel, and brexu-cel	NA	20	Any grade: 55.0 Grade ≥ 3: 5.0	All grades: 45.0 Grade ≥ 3: 25.0	Late infection events: 29.0, Hypogammaglobulinemia (IgG < 400 mg/dL or IVIg) Pre-lymphodepletion: 31.0 Late hypogammaglobulinemia: 83.0
				Axi-cel	NA	3	Grade ≥ 3: 33.0	Grade ≥ 3: 67.0	NA
				Liso-cel	NA	14	NA	Grade ≥ 3: 7.0	NA
				Brexu-cel	NA	1	NA	Grade ≥ 3: 100	NA

Abbreviations: 3L+, third line and greater; AE, adverse event; ALL, acute lymphoblastic leukemia; axi-cel, axicabtagene ciloleucel; BCL, B-cell lymphoma; brexu-cel, brexucabtagene autoleucel; CAR-T, chimeric antigen receptor T-cell; CLL, chronic lymphocytic leukemia; CRS, cytokine release syndrome; DLBCL, diffuse large B-cell lymphoma; FL, follicular lymphoma; Ig, immunoglobulin; IV, intravenous; liso-cel, lisocabtagene maraleucel; MCL, mantle cell lymphoma; NA, not available; NHL, non-Hodgkin lymphoma; Ph., phase; R/R, relapsed/refractory; tisa-cel, tisagenlecleucel; WM, Waldenstrom macroglobulinemia.

**Table 5:** Detailed response results from the studies identified.

Study	Trial Name/ID	Study Design	Patient Population	Treatment	Follow-Up	Outpatients					Inpatients				
						*n*	ORR % (95% CI)	CR % (95% CI)	PR % (95% CI)	Other Details	*n*	ORR % (95% CI)	CR % (95% CI)	PR % (95% CI)	Other Details
Linhares (2022) [37], Godwin (2021) [36]	OUTREACH	Ph 2 trial	R/R LBCL (3L+)	Liso-cel	NR	57	82.4 (70.0–91.0)	58.0	25.0	SD: 5.0% Median DOR: 15.1 (3.9–NR)	25	76.0 (55.0–91.0)	44.0	32.0	SD: 12.0; median DOR: 14.8 (2.0–NR)
Sehgal (2022) [39]	PILOT	Ph 2 trial	R/R LBCL (2L+)	Liso-cel	Median: 104.3 weeks	20	80.0 (56.3–94.3)	50.0 (27.2–72.8)	30.0	Median DOR: 8.2 (2.1–NR)	41	80.0	56.0	24.0	IP/OP (*n* = 61); SD: 5.0%; median DOR: 12.1 (6.2–NR)
Abramson (2020) [42]	TRANSCEND NHL 001	Ph 2 trial	R/R LBCL (2L+)	Liso-cel	NR	25	80.0 (59.3–93.2)	56.0 (34.9–75.6)	24.0	Median DOR: NR (2.4–NR)	231	72.0	53.0	19.0	IP/OP (*n* = 256): ORR: 73.0%, CR: 53.0%
Shadman (2021) [47]	NCT03277729	Ph 1/2 trial	R/R FL	CAR-T cells	13.0 months post-infusion	18	94.0	78.0	17.0	NR	NA	NA	NA	NA	NA
			R/R MCL, CLL, DLBCL, WM/LPL			7	100.0	57.0	43.0	NR	NA	NA	NA	NA	NA
Borogovac (2022) [57]	-	Obs. study	DLBCL and ALL	Axi-cel	1.0 month	13	77.0	69.0	8.0	PR or SD: 8.0%	NR	NR	NR	NR	NR
				Tisa-cel, brexu-cel, liso-cel		8	87.5	75.0	12.5	PR or SD: 13.0%	NR	NR	NR	NR	NR
Nasta (2022) [60]	-	Obs. Study	Lymphoma	Tisa-cel	39.5 weeks	72	43.0	34.7	8.3	SD: 5.6%	NR	NR	NR	NR	NR
Shao (2021) [57]	-	Obs. study	DLBCL	Tisa-cel	29.1 weeks	12	58.0	25.0	33.0	SD: 8.3%	NR	NR	NR	NR	NR
Shadman (2021) [47]	NCT03277729	Ph 1/2, CT	FL	CAR-T cells	13.0 months post-infusion	18	94.0	78.0	17.0	NR	NR	NR	NR	NR	NR
			DLBCL			2	100	50.0	50.0	NR	NR	NR	NR	NR	NR
			WM/LPL			2	100	100	0	NR	NR	NR	NR	NR	NR
			CLL			1	100	100	0	NR	NR	NR	NR	NR	NR
			MCL			2	100	NR	100	NR	NR	NR	NR	NR	NR
Borogovac (2022) [57]	-	Obs. Study	DLBCL and ALL	Axi-cel	1.0 month	13	77.0	69.0	8.0	PR or SD: 8.0%	NR	NR	NR	NR	NR
				Tisa-cel		6	100	83.0	17.0	PR or SD: 17.0%	NR	NR	NR	NR	NR
				Brexu-cel		1	100	100	0	PR or SD: 0.0%	NR	NR	NR	NR	NR
				Liso-cel		1	0	0	0	PR or SD: 0.0%	NR	NR	NR	NR	NR
Nasta (2022) [60]	-	Obs. Study	Lymphoma	Tisa-cel No Bridging	39.5 weeks	17	53.0	29.4	23.5	SD: 5.9%	NR	NR	NR	NR	NR
				Tisa-cel Bridging		55	40.0	36.4	3.6	SD: 5.5%	NR	NR	NR	NR	NR

Abbreviations: 2L+, second line and greater; 3L+, third line and greater; axi-cel, axicabtagene ciloleucel; brexu-cel, brexucabtagene autoleucel; CAR-T, chimeric antigen receptor T-cell; CI, confidence interval; CLL, chronic lymphocytic leukemia; CR, complete response; CT, clinical trial; DLBCL, diffuse large B-cell lymphoma; DOR, duration of response; FL, follicular lymphoma; IP, inpatient; LBCL, large B-cell lymphoma; liso-cel, lisocabtagene maraleucel; LPL, lymphoplasmacytic lymphoma; MCL, mantle cell lymphoma; NA, not applicable; NHL, non-Hodgkin lymphoma; NR, not reported; Obs., observational; OP, outpatient; ORR, overall response rate; ph, phase; PR, partial response; R/R, relapsed/refractory; RWE, real-world evidence; SD, stable disease; tisa-cel, tisagenlecleucel; WM, Waldenstrom macroglobulinemia.

**Table 6:** Progression-free survival data reported in the included studies.

Study	Trial Name/ID	Study Design	Patient Population	Treatment	Follow-Up	Outpatient				Inpatient			
						*n*	Median PFS in Months (95% CI)	PFS Rate (95% CI)	Other Data	*n*	Median PFS in Months (95% CI)	PFS Rate (95% CI)	Other Data
Linhares (2022) [37]	OUTREACH	Ph 2 trial	R/R LBCL (3L+)	Liso-cel		57	6.1 (2.9-NR)	12-mo: 41.0%	-	25	4.3 (2.8-NR)	12-mo: 39.0%	
Fowler (2023) [67]	ELARA	Ph 2 trial	R/R FL (3L+)	Tisa-cel	20.0 months	17	NA	12-mo: 60% 18-mo: 57.0%	-	80	NA	12-mo: 70.0% 18-mo: 62.5 (34.9–81.1)	-
Sehgal (2022) [39]	PILOT	Ph 2 trial	R/R LBCL (2L+)	Liso-cel	12.3 (IQR, 6.1–18.0)	20	7.2 (2.4- 13.0)	NR	Median EFS: 7.1 months (2.4–13.0)	NA	NA	NA	Overall patients (*n* = 61) Median PFS: 9.0 months (4.2–NR)
Abramson (2020) [42]	TRANSCEND NHL 001	Ph 1 trial	R/R LBCL (2L+)	Liso-cel	18.8 months	25	NR (3.0–NR)	NR	-	NA	NA	NA	Overall patients (*n* = 256) Median PFS: 6.8 months (3.3–14.1) 6-mo PFS: 51.4% (45.0–58.0) 12-mo PFS: 44.1% (37.3–50.7)
Kirby (2022) [63]	Kirby 2022	Retr. study	R/R BCL	Liso-cel, axi-cel, tisa-cel, brexu-cel	>3.3 months	20	NR	6-mo: 65.0% 12-mo: 60.0%	-	-	-	-	-

Abbreviations: 2L+, second line and greater; 3L+, third line and greater; axi-cel, axicabtagene ciloleucel; BCL, B-cell lymphoma; brexu-cel, brexucabtagene autoleucel; CI, confidence interval; EFS, event-free survival; FL, follicular lymphoma; IQR, interquartile range; LBCL, large B-cell lymphoma; liso-cel, lisocabtagene maraleucel; mo, month; *n*, total population; NA, not applicable; NR, not reached; ph, phase; PFS, progression-free survival; R/R, relapsed/refractory; Retr., retrospective; tisa-cel, tisagenlecleucel.

**Table 7:** Overall survival data reported in the included studies.

Study	Trial Name/ID	Study Design	Patient Population	Treatment	Follow-Up	Outpatient				Inpatient			
						*n*	Median OS in Months (95% CI)	OS Rate (95% CI)	Other Data	*n*	Median OS in Months (95% CI)	OS Rate (95% CI)	Other Data
Linhares (2022) [37]	OUTREACH	Ph 2 trial	R/R LBCL (3L+)	Liso-cel	-	57	NR (NR-NR)	12-mo: 60.0%	-	25	22.2 (8.0-NR)	12-mo: 60.0%	-
Sehgal (2022) [39]	PILOT	Ph 2 trial	R/R LBCL (2L+)	Liso-cel	12.3 (IQR, 6.1–18.0)	20	NR (10.5–NR)	NA	-	NA	NA	NA	Overall patients (*n* = 61): 17.6 months Median (months) (95% CI): Not reached (19.3-not reached)
Nasta (2022) [60]	Nasta 2022	Retr. study	Lymphoma	tisa-cel	9.1	72	26.5 (19.0–NR)	NA	-	-	-	-	-
Kirby (2022) [63]	Kirby 2022	Retr. study	R/R BCL	Liso-cel, axi-cel, tisa-cel, brexu-cel	>3.3 months	20	NA	6-mo: 85.0% 12-mo: 75.0%	-	-	-	-	-
Shadman (2021) [47]	NCT03277729	Ph 1/2 trial	R/R B-NHL and CLL	MB-106 (CD20 CAR-T)	8.9	25	NA	1.0	1 death over FU	-	-	-	-
			R/R FL	MB-106 (CD20 CAR-T)	9.3	18	NA	0.9	1 death over FU	-	-	-	-

Abbreviations: 2L+, second line and greater; 3L+, third line and greater; axi-cel, axicabtagene ciloleucel; BCL, B-cell lymphoma; B-NHL, B-cell non-Hodgkin lymphoma; brexu-cel, brexucabtagene autoleucel; CAR-T, chimeric antigen receptor T-cell; CI, confidence interval; CLL, chronic lymphocytic leukemia; DLBCL, diffuse large B-cell lymphoma; FL, follicular lymphoma; FU, follow-up; IQR, interquartile range; LBCL, large B-cell lymphoma; liso-cel, lisocabtagene maraleucel; mo, month; *n*, total population; NA, not available; NR, not reported; OS, overall survival; Ph, phase; PR, partial response; Retr., retrospective; R/R, relapsed/refractory; tisa-cel, tisagenlecleucel.

**Table 8:** Quality of life in the OUTREACH trial.

Study	Instruments	Domains	Outpatient (*n* = 54), LS Mean Change from Baseline (95% CI)	Inpatient (*n* = 28), LS Mean Change from Baseline (95% CI)	*p* Value
Linhares (2022) [38] (OUTREACH)	EORTC QLQ-C30	GH/QoL	7.80 (3.99–11.61)	10.39 (5.37–15.42)	0.415
		Physical functioning	0.38 (−1.11 to 4.08)	3.50 (−1.33 to 8.34)	0.312
		Role functioning	5.17 (0.27–10.01)	4.8 (−1.63 to 11.21)	0.927
		Cognitive functioning	0.71 (−1.08 to 4.51)	1.83 (−3.19 to 6.84)	0.716
		Fatigue	−6.28 (−10.73 to −1.82)	−11.18 (−16.99 to −5.36)	0.188
		Pain	−13.46 (−17.50 to −9.41)	−13.25 (−18.51 to −8.00)	0.951
	EQ-5D-5L	HUI	0.02 (−0.02 to 0.06)	0.04 (−0.01 to 0.10)	0.51
		VAS	7.48 (4.10–10.86)	10.34 (5.76–14.91)	0.31

Higher scores on GH/QoL and EQ-5D VAS reflect better QoL. Higher scores on the functioning scales (physical, role, and cognitive) indicate better QoL. Higher scores on the symptom scales (fatigue and pain) indicate worse QoL. Abbreviations: CI, confidence interval; EORTC QLQ-C30, European Organization for Research and Treatment of Cancer Quality of Life Questionnaire-Core 30; EQ-5D-5L, EuroQol 5-level 5-Dimension Questionnaire; GH, Global Health; HUI, Health Utility Index; LS, least-squares; QoL, quality of life; VAS, visual analog scale.

**Table 9:** Costs associated with patients receiving chimeric antigen receptor T-cell therapy in outpatient and inpatient settings.

First Author, Year	Trial Name/ID	Country for Cost Analysis	Study Design	Patient Population	Treatment	Cost Components	Follow-Up	Site of Care: Outpatient				Site of care: Inpatient			
								*n*	Total Costs	Hosp. Costs	Other Costs	*n*	Total Costs	Hosp. Costs	Other Costs
Palomba (2020) [52]	TRANSCEND NHL 001 and OUTREACH	US	Pooled analysis	R/R LBCL (3L+)	Liso-cel	IP and ICU LOS, diagnostics, procedures, medications	6.0 months	47	6-month post-infusion cost: USD 36,702 First month: USD 19,837	NA	NA	256	6-month post-infusion cost: USD 89,535 First month: USD 50,369	NA	NA
Denlinger (2022) [53] *	NA	US	Retrospective study	BCL	Tisa-cel; axi-cel	NA	Median: Axi-cel: 31.4 months Tisa-cel: 23.8 months	18	Tisa-cel (outpatient *): Median (range): USD 64,834 (USD 4007–USD 429,380)	NA	NA	45	Axi-cel (inpatient *): Median (range): USD 176,535 (USD 30,977–USD 1187,965)	NA	NA
McGarvey (2022) [41]	PILOT/NCT03483103	US	Ph 2 trial	R/R LBCL (2L+) not intended for HSCT	Liso-cel	IP and ICU LOS, diagnostics, procedures, medications	NA	20	6-month post-infusion monitoring cost: USD 16,172 First month: USD 13,261	USD 9455	NA	41	6-month post-infusion monitoring cost: USD 61,772 First month: USD 46,947	USD 49,495	NA
McGarvey (2022) [65]	TRANSFORM/NCT03575351	US	Ph 3 trial	R/R LBCL (2L+)	Liso-cel	IP and ICU LOS, diagnostics, procedures, medications	NA	19	6-month post-infusion monitoring cost: USD 38,314 First month: USD 18,774	USD 20,867	NA	71	6-month post-infusion monitoring cost: USD 96,297 First month: USD 49,111	USD 69,153	NA
Fowler (2023) [67]	ELARA/NCT03568461	US	Ph 2 trial	R/R FL (3L+)	Tisa-cel	IP and ICU LOS	Median: 20.0 months	17	NA	USD 7477	NA	80	NA	USD 40,054	NA
Maziarz (2022) [62]	NA	US	Retrospective study	R/R DLBCL	Tisa-cel, axi-cel	IP and OP	Mean: 5.0 months	Tisa-cel: 8	For FU period: Tisa-cel: USD 13,389 For infusion encounter: Tisa-cel: USD 4741	Tisa-cel: USD 4753	OP costs: Tisa-cel: USD 8636	Tisa-cel: 25 Axi-cel: 86	For FU period: Axi-cel: USD 46,575 Tisa-cel: USD 33,701 Infusion encounter: Axi-cel: USD 51,378 Tisa-cel: USD 34,908	For FU period: Axi-cel: USD 44,561 Tisa-cel: USD 29,953	For FU period: OP costs: Axi-cel: USD 2014 Tisa-cel: USD 3748
Yang (2022) [26] **	NA	US	RWE	R/R DLBCL	Tisa-cel, axi-cel	IP, ER, OP, other medical services, medications	CAR-T IP: 6.6 months, CAR-T OP: 6.0 months	50	Total Medicare reimbursement amounts for months 1, 2, 3, 4, 5, 6, and 7: USD 371,839, USD 9120, USD 4927, USD 8300, USD 8102, USD 6724, and USD 10,883, respectively	NA	NA	380	Total Medicare reimbursement amounts for months 1, 2, 3, 4, 5, 6, and 7: USD 348,364, USD 9756, USD 7318, USD 8259, USD 7052, USD 5748, and USD 6741, respectively	NA	NA

* Denlinger et al. did not specify the settings; however, it seems that tisa-cel was administered in the outpatient setting and axi-cel in the inpatient setting. ** Reimbursement amounts were assessed only among patients who received CAR-T in non-prospective payment system-exempt hospitals. Abbreviations: 2L+, second line and greater; 3L+, third line and greater; axi-cel, axicabtagene ciloleucel; BCL, B-cell lymphoma; CAR-T, chimeric antigen receptor T-cell; DLBCL, diffuse large B-cell lymphoma; ER, emergency room; FL, follicular lymphoma; FU, follow-up; hosp., hospitalization; ICU, intensive care unit; IP, inpatient; LBCL, large B-cell lymphoma; LOS, length of stay; NA, not available; OP, outpatient; Ph, phase; RWE, real-world evidence; R/R, relapsed/refractory; tisa-cel, tisagenlecleucel; US, United States.

**Table 10:** Healthcare resource utilization associated with patients receiving chimeric antigen receptor T-cell therapy in the outpatient and inpatient settings.

Study Details	Treatment	Outpatient Cohort							Inpatient Cohort					
		*n*	OP Visits	Hospitalization Rate	Time to Hospitalization	Reasons for Hospitalization	LOS	ICU Admissions	*n*	OP Visits	Hospitalization Rate	Time to Hospitalization	LOS	ICU Admissions
Chihara (2022) [54]	Any CAR-T therapy	95	NA	Follow-up hospitalization: 41.8%	NA	NA	NA	NA	456	NA	Within 90 days: rehospitalization: 28.7%	NA	21.4 days	NA
Wright (2020) [68]	Tisa-cel, axi-cel	12	NA	Unplanned hospitalization: 33.0%	NA	NA	NA	NA	19	NA	Unplanned hospitalization: 26.0%	NA	NA	NA
Palomba (2020) [52]	Liso-cel	47	100%	62.0%	NA	NA	Total: 7.8 (SD, 13.1) days ICU: 0.6 (SD, 2.8) days	6.0%	256	93.0%	~100%	NA	Total: 20.1 (SD, 15.1) days ICU: 1.1 (SD, 5.5) days	7.0%
Sehgal (2022) [39]	Liso-cel	20	NA	Total: 45.0% Within 72 h: 10.0%	Median: 6.0 (IQR, 5.0–10.0) days	AEs: 78.0% Other: 22.0%	Initial hospitalization: Mean: 2.5 (SD, 3.2) days Median: 5.0 (IQR, 4.0–7.0) days ICU: Mean: 3.0 days Median: 3.0 (IQR, 3.0–3.0) days	5.0%	41	NA	Total: 100%	NA	Initial hospitalization: Mean: 11.9 (SD, 5.1) days Median: 12.0 (8.0–15.0) days ICU: Mean: 2.5 (SD, 0.7) days Median: 2.5 (IQR, 2.0–3.0) days	20.0%
Yang (2022) [26]	Tisa-cel, axi-cel	50	NA	Within the 1st month: 52.0%	NA	NA	LOS for months 1, 2, 3, 4, 5, 6, and 7: 5.2, 1.6, 1.2, 1.3, 1.4, 0.7, and 0.9 days, respectively ICU LOS: for months 1, 2, 3, 4, 5, 6, and 7: 0.6, 0.1, 0.1, 0.3, 0, 0, and 0.1 days, respectively	NA	380	NA	Within the 1st month: 100% (by definition)	NA	LOS for months 1, 2, 3, 4, 5, 6 and 7: 20.4, 4.6, 2.4, 2.0, 1.4, 1.1 and 1.0 days, respectively ICU LOS: for months 1, 2, 3, 4, 5, 6 and 7: 2.4, 0.1, 0.1, 0.1, 0.1, 0.1, and 0.1 days, respectively	NA
Fowler (2023) [67]	Tisa-cel	17	NA	59.0%	5.8 (SD, 7.1) days	CRS: 53.0%	Total: Mean: 4.3 (SD, 1.4) days Median: 4.5 days ICU: 0.0 days	0.0%	80	NA	100%	NA	Total: Mean: 13.8 (SD, 8.5) days Median: 12.5 days ICU: Median: 4.0 days	9.0%
Linhares (2022) [37]	Liso-cel	54	NA	Overall hospitalization: 76.0% Within 4 days: 31.0%	Median: 5.0 days (2.0–310.0 days)	AEs: 83.0% Other: 17.0%	Initial hospital stay: Median: 6.0 (1.0–28.0) days ICU: Median: 3.5 (2.0–5.0) days	4.0%	25	NA	100%	NA	Initial hospital stay: Median initial stay: 13.0 days (1.0–31.0)	NA
McGarvey (2022) [65]	Liso-cel	19	NA	NA	NA	NA	Median: 9.0 (range, 4.0–33.0) days	NA	71	NA	NA	NA	Median: 15.0 (range, 1.0–164.0) days	NA
Denlinger (2022) [53]	Tisa-cel, axi-cel	Tisa-cel: 18	NA	NA	NA	NA	Tisa-cel: 9.0 days	NA	Axi-cel: 45	NA	NA	NA	Axi-cel: 14.0 days	NA
Maziarz (2022) [61]	Tisa-cel, axi-cel	Tisa-cel: 8	100%	63.0%	NA	NA	Mean: 1.7 days ICU: 0.4 days	38.0%	Axi-cel: 86 Tisa-cel: 25	Axi-cel: 52.0% Tisa-cel: 76.0%	NA	NA	Axi-cel: 6.9 days Tisa-cel: 4.6 days	Axi-cel: 30.0% Tisa-cel: 16.0%
Myers (2020) [46]	Tisa-cel	198 *	NA	Within 30 days: 70.0%	NA	NA	Median: 7.0 (IQR, 4.0–13.0) days	23.0%	15 *	NA	NA	NA	NA	NA
Kamdar (2022) [64]	Liso-cel	19	NA	68.0%	Median: 9.0 (IQR, 4.0–19.0) days	CRS: 38.0% Other AEs: 38.0% PD: 8.0% Other: 15.0%	Median: 9.0 (IQR, 5.0–9.0) days	0.0%	NA	NA	NA	NA	NA	NA
Kirby (2022) [63]	Tisa-cel, axi-cel, liso-cel, brexu-cel	20	NA	Within 1st month of therapy: Overall: 50.0% Axi-cel: 67.0% Liso-cel: 36.0% Tisa-cel: 100% Brexu-cel: 100%	NA	NA	NA	NA	NA	NA	NA	NA	NA	NA
Nasta (2022) [60]	Tisa-cel	68	NA	Within 72 h: 19.4% Within 30 days: 36.1%	NA	CRS: 85% Infection: 8% Colitis: 4% Catatonia: 4%	Median: 5.0 days	NA	4	NA	NA	NA	NA	NA
Shao (2021) [57]	Tisa-cel	12	NA	Within 30 days: 50.0%	Median: 4.0 days (2.0–12.0 days)	CRS: 83.0% Colitis: 17.0%	Median: 5.5 days (2.0–9.0) days	NA	NA	NA	NA	NA	NA	NA
Borogovac (2022) [55]	Axi-cel, tisa-cel, brexu-cel, liso-cel	21	NA	Post therapy: Within 72 h/within 1st month: Overall: 24.0%/71.0% Axi-cel: 23.0%/76.0% Liso-cel: 0.0%/0.0% Tisa-cel: 33.0%/67.0% Brexu-cel: 0.0%/100%	Median: 4.0 (1.0–28.0) days	Fever: 87.0% CNS toxicity: 13.0%	Median: 8.0 days (1.0–30.0)	NA	2	NA	NA	NA	NA	NA
Farooqui (2022) [59]	Axi-cel	With AKI: 14 Without AKI: 69	NA	NA	NA	NA	NA	With AKI: 42.9% Without AKI: 29.0%	NA	NA	NA	NA	NA	NA

* In Myers et al., 93% of all patients (198 of 213) received CAR-T in the outpatient setting. Hence, these data were reported under the outpatient cohort. Abbreviations: AE, adverse event; AKI, acute kidney injury; axi-cel, axicabtagene ciloleucel; brexu-cel, brexucabtagene autoleucel; CAR-T, chimeric antigen receptor T-cell; CNS, central nervous system; CRS, cytokine release syndrome; ER, emergency room; ICU, intensive care unit; IQR, interquartile range; liso-cel, lisocabtagene maraleucel; LOS, length of stay; NA, not available; OP, outpatient; PD, progressive disease; SD, standard deviation; tisa-cel, tisagenlecleucel.

## Data Availability

All data generated or analyzed during this study are included in this published article (and its Supplementary Information Files).

## Supplementary Materials

The following supporting information can be downloaded at https://www.mdpi.com/article/10.3390/cancers15245746/s1. Table S1. MEDLINE search strategy; Table S2. Embase search strategy; Table S3. Cochrane search strategy; Table S4. Quality assessment of randomized controlled trial using the Risk-of-Bias version 2 checklist; Table S5. Quality assessment of non-randomized clinical trials (single-arm) using Downs and Black checklist; Table S6. Quality assessment of observational studies (single-arm and double-arm) using the NOS checklist; Table S7. Additional healthcare resource utilization data available in identified studies.
