# Supplementary material for: The Impact of Outpatient versus Inpatient Administration of CAR-T Therapies on Clinical, Economic, and Humanistic Outcomes in Patients with Hematological Cancer: A Systematic Literature Review
Source: Cancers (Basel). 2023 Dec 7;15(24):5746. doi: 10.3390/cancers15245746 (PMC10741664; doi:10.3390/cancers15245746)
Supplement: Supplementary file 1 [file cancers-15-05746-s001.zip › cancers-2738340-supplementary.pdf]

Type of the paper: Systematic Review

The Impact of Outpatient versus Inpatient Administration of CAR-T Therapies on Clinical, Economic, and Humanistic Outcomes in Patients with Hematological Cancer: A Systematic Literature Review

Supplementary materials

Table S1. MEDLINE search strategy

#	Query	# hits
1	exp Lymphoma, B-Cell/	54,269
2	exp Lymphoma, Non-Hodgkin/	111,877
3	(non hodgkin* or nonhodgkin* or nhl).ti,ab.	42,633
4	(diffus* adj3 lymphom*).ti,ab.	4859
5	exp Lymphoma, Large B-Cell, Diffuse/	23,022
6	((large cell* adj3 lymphom*) or (b cell adj3 lymphom*) or (bcell adj3 lymphom*)).ti,ab.	50,665
7	DLBCL.ti,ab.	9404
8	exp Lymphoma, Follicular/	6776
9	exp Lymphoma, Mantle-Cell/	3646
10	or/1-9	145,880
11	exp acute lymphoblastic leukemia/	33,119
12	acute B cell leukemia.ti,ab.	29
13	(acute adj3 (lymphocyte or lymphocytic or lymphatic or lymphoblastic or lymphoid or lymphocyte or T cell or B cell) adj (leukemia or leukaemia)).ti,ab.	42,389

14	or/11-13	51,551
15	exp multiple myeloma/	46,762
16	(myeloma* or (multiple adj myeloma*) or plasmacytom* or plasmocytom* or (monoclonal adj gammopath*)).ti,ab.	69,448
17	or/15-16	77,163
18	10 or 14 or 17	263,844
19	((chimeric* adj3 antigen receptor*) or (chimeric* adj3 immunoreceptor*) or (chimeric* adj3 T cell receptor*) or (chimeric* adj3 Tcell receptor*)).mp. and (therap* or treat* or immunity or immunotherap* or cell*).ti,ab.	8603
20	((artificial* adj3 T cell receptor*) or (artificial* adj3 Tcell receptor*)).ti,ab.	12
21	axicabtagene*.ti,ab.	316
22	(yescarta* or "KTE-C19" or "CTL 019").ti,ab.	99
23	(kymriah* or "CART-19" or "CART19").ti,ab.	160
24	Lisocabtagene*.ti,ab.	59
25	(lisocel* or "JCAR017").ti,ab.	2
26	(Abecma or idecabtagene vicleucel).ti,ab.	49
27	(Tecartus or brexucabtagene autoleucel).ti,ab.	43
28	(Carvykti or Ciltacabtagene autoleucel).ti,ab.	31
29	or/19-28	8730
30	18 and 29	2872
31	exp animals/ not humans/	5,086,122
32	(comment or letter or editorial or "case reports").pt.	4,206,817
33	(case stud$ or case report$).ti.	365,015
34	(address or autobiography or biography or case reports or veterinary trials or veterinary as topic or comment or dictionary or directory or duplicate publication or editorial or festschrift or guideline or historical article or interactive tutorial or interview or lecture or legislation or letter or	7,549,475

	observational study, veterinary or patient education handout or personal narrative or practice guideline or editorial or erratum or letter or note or review or short survey or comment*).pt.	
35	or/31-34	12,435,750
36	30 not 35	1432
37	limit 36 to yr="2016 -Current"	**1341**

Table S2. Embase search strategy

#	Query	# hits
1	exp B cell lymphoma/	140,481
2	exp nonhodgkin lymphoma/	195,203
3	(non hodgkin* or nonhodgkin* or nhl).ti,ab.	67,971
4	(diffus* adj3 lymphom*).ti,ab.	7563
5	exp diffuse large B cell lymphoma/	23,176
6	((large cell* adj3 lymphom*) or (b cell adj3 lymphom*) or (bcell adj3 lymphom*)).ti,ab.	78,546
7	DLBCL.ti,ab.	22,876
8	exp follicular lymphoma/	19,334
9	exp mantle cell lymphoma/	14,033
10	or/1-9	274,152
11	exp acute lymphoblastic leukemia/	66,948
12	acute B cell leukemia.ti,ab.	40
13	(acute adj3 (lymphocyte or lymphocytic or lymphatic or lymphoblastic or lymphoid or lymphocyte or T cell or B cell) adj (leukemia or leukaemia)).ti,ab.	63,330
14	or/11-13	84,175
15	exp multiple myeloma/	92,500
16	(myeloma* or (multiple adj myeloma*) or plasmacytom* or plasmocytom* or (monoclonal adj gammopath*)).ti,ab.	108,081

17	or/15-16	126,976
18	10 or 14 or 17	449,450
19	((chimeric* adj3 antigen receptor*) or (chimeric* adj3 immunoreceptor*) or (chimeric* adj3 T cell receptor*) or (chimeric* adj3 Tcell receptor*)).mp. and (therap* or treat* or immunity or immunotherap* or cell*).ti,ab.	22,749
20	((artificial* adj3 T cell receptor*) or (artificial* adj3 Tcell receptor*)).ti,ab.	43
21	axicabtagene*.ti,ab.	1059
22	(yescarta* or "KTE-C19" or "CTL 019").ti,ab.	234
23	(kymriah* or "CART-19" or "CART19").ti,ab.	473
24	Lisocabtagene*.ti,ab.	220
25	(lisocel* or "JCAR017").ti,ab.	41
26	(Abecma or idecabtagene vicleucel).ti,ab.	164
27	(Tecartus or brexucabtagene autoleucel).ti,ab.	97
28	(Carvykti or Ciltacabtagene autoleucel).ti,ab.	115
29	or/19-28	23,019
30	18 and 29	10,115
31	exp animals/ not humans/	11,519,325
32	(comment or letter or editorial or "case reports").pt.	1,999,682
33	(case stud$ or case report$).ti.	444,152
34	(address or autobiography or biography or case reports or veterinary trials or veterinary as topic or comment or dictionary or directory or duplicate publication or editorial or festschrift or guideline or historical article or interactive tutorial or interview or lecture or legislation or letter or observational study, veterinary or patient education handout or personal narrative or practice guideline or editorial or erratum or letter or note or review or short survey or comment*).pt.	6,549,270
35	or/31-34	16,963,668
36	30 not 35	6184
37	limit 36 to yr="2016 -Current"	**6117**

Table S3. Cochrane search strategy

#	Query	# hits
#1	[mh "Lymphoma, B-Cell"]	784
#2	[mh "Lymphoma, Non-Hodgkin"]	2215
#3	(non hodgkin* or nonhodgkin* or nhl):ti,ab	4022
#4	(diffus* NEAR/3 lymphom*):ti,ab	283
#5	[mh "Lymphoma, Large B-Cell, Diffuse"]	491
#6	((large cell* NEAR/3 lymphom*) or (b cell NEAR/3 lymphom*) or (bcell NEAR/3 lymphom*)):ti,ab	2877
#7	DLBCL:ti,ab	1242
#8	[mh "Lymphoma, Follicular"]	356
#9	[mh "Lymphoma, Mantle-Cell"]	163
#10	#1 or #2 or #3 or #4 #5 or #6 or #7 or #8 or #9	6804
#11	[mh "Precursor Cell Lymphoblastic Leukemia-Lymphoma"]	1240
#12	(acute B cell leukemia):ti,ab	858
#13	(acute NEAR/3 (lymphocyte or lymphocytic or lymphatic or lymphoblastic or lymphoid or lymphocyte or T cell or B cell) NEAR/2 (leukemia or leukaemia)):ti,ab	3198
#14	#11 or #12 or #13	3795
#15	[mh "Multiple Myeloma"]	1819
#16	(myeloma* or (multiple adj myeloma*) or plasmacytom* or plasmocytom* or (monoclonal adj gammopath*)):ti,ab	6010
#17	#15 or #16	6177
#18	#10 or #14 or #17	16,044
#19	((chimeric* NEAR/3 antigen receptor*) or (chimeric* NEAR/3 immunoreceptor*) or (chimeric* NEAR/3 T cell receptor*) or (chimeric* NEAR/3 Tcell receptor*)).mp. and (therap* or treat* or immunity or immunotherap* or cell*):ti,ab	12,600

#20	((artificial* NEAR/3 T cell receptor*) or (artificial* NEAR/3 T cell receptor*)):ti,ab	0
#21	axicabtagene*:ti,ab	44
#22	(yescarta* or "KTE-C19" or "CTL 019"):ti,ab	21
#23	(kymriah* or "CART-19" or "CART19"):ti,ab	14
#24	Lisocabtagene*:ti,ab	10
#25	(lisocel* or "JCAR017"):ti,ab	11
#26	(Abecma or idecabtagene vicleucel):ti,ab	5
#27	(Tecartus or brexucabtagene autoleucel):ti,ab	1
#28	(Carvykti or Ciltacabtagene autoleucel):ti,ab	7
#29	#19 or #20 or #21 or #22 or #23 or #24 or #25 or #26 or #27 or #28	12,691
#30	#18 and #29	468
#31	[mh "animals"] NOT [mh "humans"]	13
#32	(comment or letter or editorial or "case reports"):pt	16,664
#33	(case stud$ or case report$):ti	315
#34	(address or autobiography or biography or case reports, veterinary trials, veterinary as topic or comment or dictionary or directory or duplicate publication or editorial or festschrift or guideline or historical article or interactive tutorial or interview or lecture or legislation or letter or observational study, veterinary or patient education handout or personal narrative or practice guideline or review or editorial or erratum or letter or note or short survey or comment*):pt	29,753
#35	#31 or #32 or #33 or #34	30,076
#36	#30 not #35	467
#37	#36 with Cochrane Library publication date from Jan 2016 to Jan 2023	**243**

Table S4. Quality assessment of randomized controlled trial using the Risk-of-Bias version 2 checklist

**Study**		**Kamdar (2022)**
**Q1**	Bias arising from the randomization process	**Low risk of bias**: A permuted-blocks method with an interactive response technology system was used to minimize selection bias
**Q2**	Bias due to deviations from intended interventions	**High risk of bias**: Open label (individuals who took part in the trial were all aware of the intervention that was provided to study participants)

**Q3**	Bias due to missing outcome data	**Low risk of bias**: The study authors utilized intention-to-treat analysis to minimize attrition bias by taking into account missing patient data in the analysis
**Q4**	Bias in measurement of the outcome	**Low risk of bias**: Blinded reviews of imaging data by independent review committee was used to measure outcomes, and appropriate units [HR, N (%), etc.] were used for outcome representation
**Q5**	Bias in selection of the reported result	**Low risk of bias**: There was no reporting bias from selective reporting of results because the authors studied all the outcomes in accordance with the methodology/protocol
**Overall bias**		**High risk of bias**

Abbreviation: HR, hazard ratio

Table S5. Quality assessment of non-randomized clinical trials (single-arm) using Downs and Black checklist

**Study Name**			**Fowler (2023)**		**Sehgal (2022)**		**Abramson (2020)**		**Linhares (2022)**		**Shadman (2021)**		**Turtle (2017)**		**Myers (2020) NCT02906371**		**Myers (2020) NCT02374333**	
**Domains**	**#**	**Items**	**Score**	**Yes/No/CD**	**Score**	**Yes/No/CD**	**Score**	**Yes/No/CD**	**Score**	**Yes/No/CD**	**Score**	**Yes/No/CD**	**Score**	**Yes/No/CD**	**Score**	**Yes/No/CD**	**Score**	**Yes/No/CD**
**Reporting (10 items)**	1	Hypothesis/aim/objective clearly described?	1	Yes	1	Yes	1	Yes	1	Yes	1	Yes	1	Yes	1	Yes	1	Yes
	2	Main outcomes in Introduction or Methods	1	Yes	1	Yes	1	Yes	1	Yes	1	Yes	1	Yes	1	Yes	1	Yes
	3	Patient characteristics clearly described?	1	Yes	1	Yes	1	Yes	1	Yes	1	Yes	1	Yes	1	Yes	1	Yes
	4	Interventions of interest clearly described?	1	Yes	1	Yes	1	Yes	1	Yes	1	Yes	1	Yes	1	Yes	1	Yes
	5	Principal confounders clearly described?	NA	NA	NA	NA	NA	NA	NA	NA	NA	NA	NA	NA	NA	NA	NA	NA
	6	Main findings clearly described?	1	Yes	1	Yes	1	Yes	1	Yes	1	Yes	1	Yes	1	Yes	1	Yes
	7	Estimates of random variability provided for the main outcomes?	1	Yes	1	Yes	1	Yes	1	Yes	0	No	0	No	1	Yes	1	Yes
	8	All adverse events of intervention reported?	1	Yes	1	Yes	1	Yes	1	Yes	1	Yes	1	Yes	1	Yes	1	Yes
	9	Characteristics of patients lost to follow-up described?	1	Yes	1	Yes	0	No	0	No	1	Yes	0	No	0	No	0	No
	10	Probability values reported for main outcomes?	0	No	0	No	0	No	0	No	0	No	0	No	1	Yes	1	Yes
**External validity (3 items)**	11	Subjects asked to participate were representative of source population?	0	CD	0	CD	0	CD	0	CD	0	CD	0	CD	0	CD	0	CD
	12	Subjects prepared to participate were representative of source population?	0	CD	0	CD	0	CD	0	CD	0	CD	0	CD	0	CD	0	CD
	13	Location and delivery of study treatment was representative of source population?	1	Yes	0	CD	1	Yes	1	Yes	1	Yes	1	Yes	1	Yes	1	Yes
**Internal validity - bias (7 items)**	14	Study participants blinded to treatment	NA	NA	NA	NA	NA	NA	NA	NA	NA	NA	NA	NA	NA	NA	NA	NA
	15	Blinded outcome assessment?	NA	NA	NA	NA	NA	NA	NA	NA	NA	NA	NA	NA	NA	NA	NA	NA
	16	Any data dredging clearly described?	1	Yes	1	Yes	1	Yes	1	Yes	1	Yes	1	Yes	1	Yes	1	Yes

	17	Analyses adjusted for differing lengths of follow-up	NA	NA	NA	NA	NA	NA	NA	NA	NA	NA	NA	NA	NA	NA	NA	NA
	18	Appropriate statistical tests performed?	1	Yes	1	Yes	1	Yes	0	No	0	No	0	No	1	Yes	1	Yes
	19	Compliance with the intervention was reliable?	1	Yes	1	Yes	1	Yes	1	Yes	1	Yes	1	Yes	1	Yes	1	Yes
	20	Outcome measures were reliable and valid?	1	Yes	1	Yes	1	Yes	1	Yes	1	Yes	1	Yes	1	Yes	1	Yes
**Confounding (selection bias)- (6 items)**	21	All participants recruited from the same source population?	NA	NA	NA	NA	NA	NA	NA	NA	NA	NA	NA	NA	NA	NA	NA	NA
	22	All participants recruited over the same time period?	NA	NA	NA	NA	NA	NA	NA	NA	NA	NA	NA	NA	NA	NA	NA	NA
	23	Participants randomized to intervention groups?	NA	NA	NA	NA	NA	NA	NA	NA	NA	NA	NA	NA	NA	NA	NA	NA
	24	Allocation of treatment concealed from intervention and participants?	NA	NA	NA	NA	NA	NA	NA	NA	NA	NA	NA	NA	NA	NA	NA	NA
	25	Adequate adjustment for confounding?	0	CD	1	Yes	0	CD	0	CD	0	CD	0	CD	0	CD	0	CD
	26	Losses to follow-up taken into account?	1	Yes	1	Yes	0	No	0	No	1	Yes	0	No	0	CD	0	No
**Power (1 item)**	27	Sufficient power to detect treatment effect at significance level of 0.05?	0	CD	1	Yes	0	CD	0	CD	0	CD	0	CD	0	CD	0	CD
**Total score (n/N)**			**14/19**		**15/19**		**12/19**		**11/19**		**12/19**		**10/19**		**13/19**		**13/19**	
**Overall quality**			**Fair**		**Good**		**Fair**		**Poor**		**Fair**		**Poor**		**Fair**		**Fair**	

Abbreviations: CD, can't determine; NA, not applicable

Table S6. Quality assessment of observational studies (single-arm and double-arm) using the NOS checklist

**Study ID**			**Gofshteyn (2018)***	**Farooqui (2022)**	**Nasta (2022)***	**Maziarz (2022)**	**Borogovac (2022)***	**Yang (2022)**	**McGarvey (2022)***	**Shao (2021)***	**Denlinger (2022)**	**Chihara (2022)***	**Kirby (2022)**	**Wright (2020)**
Selection	Q1	Selection of the exposed cohort	1	1	1	1	0	1	1	1	1	1	1	1
		Justification	Enrolled from University of Pennsylvania	Recruited from Mayo Clinic in Rochester	Enrolled from University of Pennsylvania	Details obtained from the large service-level premier database for US hospitals and hospital-affiliated entities	Enrolled from University of Oklahoma Health Sciences Center	Enrolled from Centers for Medicare & Medicaid Services - claims database	Enrolled from TRANSFORM trial	Enrolled from Karmanos cancer center	Enrolled from Ohio State University Wexner Medical Centre	Enrolled from administrative medical and pharmacy claims Medicare fee-for-service database	Recruited from LDS hospital	Enrolled from University of Pennsylvania
	Q2	Selection of non-exposed cohort	0	1	0	1	0	1	0	0	1	0	1	1
		Justification	NA	Recruited from Mayo Clinic in Rochester	NA	Details obtained from the large service-level premier database for US hospitals and	NA	Enrolled from Centers for Medicare & Medicaid Services–claims database	NA	NA	Enrolled from Ohio State University Wexner Medical Centre	NA	Recruited from LDS hospital	Enrolled from University of Pennsylvania

						hospital-affiliated entities								
	Q3	Exposure assessment	1	1	0	1	1	1	1	1	1	1	0	0
		Justification	All data obtained through the NCT01626495 investigators and EMR	EMR	No such information provided	Hospital records (premier database)	Hospital records	Medicare & Medicaid Services–claims database	Patient case reports (TRANSFORM trial)	Chart records	Hospital records	Medicare service database	No information provided	No information provided
	Q4	Outcome is not present at start of study	1	1	1	1	1	1	1	1	1	1	1	1
		Justification	Not present	Not present	Not present	Not present	Not present	Not present	Not present	Not present	Not present	Not present	Not present	Not present
Comparability	Q5	Single factor	0	0	0	0	0	1	0	0	0	0	0	0
		Justification	NA	No confounder was adjusted	NA	NA	NA	Patient age at the index date, sex, race (White vs non-White race)	NA	NA	No confounder was adjusted	NA	No confounder was adjusted	No confounder was adjusted
	Q6	Multiple factors	0	0	0	1	0	1	0	0	0	0	0	0
		Justification	NA	No confounder was adjusted	NA	Age and comorbidity index details were comparable between two groups	NA	Patient age at the index date, sex, race (White vs non-White race)	NA	NA	No confounder was adjusted	NA	No confounder was adjusted	No confounder was adjusted
Outcome/ exposure	Q7	Assessment of outcome	1	1	1	1	1	1	1	1	0	1	0	1
		Justification	EMR	Abstracted from the immune effector cell compliance program database and EMR	Hospital records	Premier database	Hospital records	Medicare claims database	Peer-reviewed literature and TRANSFORM case reports	Chart records	No information	Medicare service database	No information provided	Hospital records

	Q8	Follow-up long enough for outcomes to occur	1	1	1	1	0	1	1	1	1	1	1	1
		Justification	2 months	31 days	9.1 months	Tisa-cel: 5.0 and axi-cel: 3.3 months	No information	6.6 months	6 months	6.7 months	Axi-cel: 31.4 and tisa-cel: 23.8 months	3 months	100 days	36 days
	Q9	Adequacy of follow-up	1	1	1	1	0	0	1	1	1	1	1	1
		Justification	Loss to follow up was 0%	Loss to follow up was 0%	Loss to follow up was 0%	Loss to follow up was 0%	No information	No information	Loss to follow up was 0%	Loss to follow up was 0%	Loss to follow up was 0%	Loss to follow up was 0%	Loss to follow up was 10%	Loss to follow up was 0%
**Total score (n/N)**			6/6	7/9	5/6	8/9	3/6	8/9	6/6	6/6	6/9	6/6	5/9	6/9
**Overall quality**			Good	Good	Good	Good	Poor	Good	Good	Good	Good	Good	Poor	Good

*single arm

Single arm: Considered Q1, Q3-Q4, Q7-Q9 for QA; double arm: Considered Q1-Q9 for QA

Abbreviations: axi-cel, axicabtagene ciloleucel; EMR, electronic medical records; NA, not applicable; NOS, Newcastle-Ottawa Scale; QA, quality assessment; tisa-cel, tisagenlecleucel; US, United States

Table S7. Additional healthcare resource utilization data available in identified studies

**Study details**	**Outpatient cohort**					**Inpatient cohort**				
	**N**	**IP admissions**	**IP days**	**OP visits**	**ER visits**	**N**	**IP admissions**	**IP days**	**OP visits**	**ER visits**
Yang, 2022	50	For months 1, 2, 3, 4, 5, 6, and 7: 0.73, 0.36, 0.18, 0.26, 0.23, 0.18, and 0.17, respectively	For months 1, 2, 3, 4, 5, 6, and 7: 5.16, 1.59, 1.19, 1.34, 1.35, 0.69, and 0.86, respectively	For months 1, 2, 3, 4, 5, 6, and 7: 5.22, 3.20, 3.10, 3.24, 3.15, 3.24, and 2.65, respectively	For months 1, 2, 3, 4, 5, 6, and 7: 0.32, 0.14, 0.03, 0.12, 0.20, 0.18, and 0.14, respectively	380	For months 1, 2, 3, 4, 5, 6, and 7: 1.44, 0.47, 0.31 0.33, 0.21, 0.18, and 0.16, respectively	For months 1, 2, 3, 4, 5, 6, and 7: 20.35, 4.64, 2.42, 2.02, 1.43, 1.1, and 1.04, respectively	For months 1, 2, 3, 4, 5, 6, and 7: 2.09, 3.81, 3.27, 3.12, 2.86, 2.82, and 2.42, respectively	For months 1, 2, 3, 4, 5, 6, and 7: 0.03, 0.10, 0.13, 0.11, 0.10, 0.11, and 0.07, respectively
Chihara, 2022	Age (65–69): 24	90 days F-U Age (65–69): 1.4	NA	90 days	90 days	Age (65–69): 144	90 days Rehospitalization:	NA	90 days	90 days

	Age (70–74): 18<br>Age (75+): 37<br>Total: 79	Age (70–74): 1.3<br>Age (75+): 1.4<br>Total: 1.4		Age (65–69): 21.8<br>Age (70–74): 17.9<br>Age (75+): 17.6<br>Total: 19	Age (65–69): 1.8<br>Age (70–74): 2.3<br>Age (75+): 1.9<br>Total: 1.9	Age (70–74): 125<br>Age (75+): 97<br>Total: 366	Age (65–69): 1.3<br>Age (70–74): 1.7<br>Age (75+): 1.5<br>Total: 1.5		Age (65–69): 17.7<br>Age (70–74): 18<br>Age (75+): 16.3<br>Total: 17.4	Age (65–69): 1.6<br>Age (70–74): 1.7<br>Age (75+): 1.7<br>Total: 1.6
Maziarz, 2022	Tisa-cel: 8	For FU period: Tisa-cel: 0.33	NA	For FU period: Tisa-cel: 4.1	For FU period: Tisa-cel: 0.07	Axi-cel: 86<br>Tisa-cel: 25	For FU period:<br>Axi-cel: 0.46<br>Tisa-cel: 0.34	NA	For FU period:<br>Axi-cel: 1.17<br>Tisa-cel: 1.54	For FU period:<br>Axi-cel: 0.09<br>Tisa-cel: 0.03

Abbreviations: axi-cel, axicabtagene ciloleucel; ER, emergency room; FU, follow-up; IP, inpatient; NA, not applicable; OP, outpatient; tisa-cel, tisagenlecleucel
